# Pulse, Shunt and Storage: Hydrological Contraction Shapes Processing and Export of Particulate Organic Matter in River Networks

**DOI:** 10.1007/s10021-022-00802-4

**Published:** 2022-12-12

**Authors:** Núria Catalàn, Rubén del Campo, Lauren Talluto, Clara Mendoza-Lera, Giulia Grandi, Susana Bernal, Daniel von Schiller, Gabriel Singer, Enrico Bertuzzo

**Affiliations:** 1 Catalan Institute for Water Research (ICRA), Emili Grahit 101, 17003 Girona, Spain; 2 Laboratoire des Sciences du Climat et de l’Environnement, LSCE, CEA, CNRS, UVSQ, 91191 Gif-sur-Yvette, France; 3 Department of Ecology, University of Innsbruck, Technikerstrasse 25, 6020 Innsbruck, Austria; 4 Leibniz-Institute of Freshwater Ecology and Inland Fisheries (IGB), Müggelseedamm 310, 12587 Berlin, Germany; 5 Institute of Environmental Sciences, University of Koblenz-Landau, 76829 Landau, Germany; 6 Department of Environmental Sciences, Informatics and Statistics, University of Venice Ca ’Foscari, 30170 Venice, Italy; 7 Integrative Freshwater Ecology Group, Center for Advanced Studies of Blanes (CEAB-CSIC), C/Accés Cala St. Francesc 14, 17300 Blanes, Spain; 8 Department of Evolutionary Biology, Ecology and Environmental Sciences, Faculty of Biology, University of Barcelona, Av Diagonal, 643, 08028 Barcelona, Spain; 9 Institut de Recerca de l’Aigua (IdRA), Universitat de Barcelona (UB), Barcelona, Spain

**Keywords:** leaf litter, stream, catchment, organic carbon, organic matter degradation, carbon cycling, preconditioning, flow intermittence, modelling

## Abstract

Streams and rivers act as landscape-scale bioreactors processing large quantities of terrestrial particulate organic matter (POM). This function is linked to their flow regime, which governs residence times, shapes organic matter reactivity and controls the amount of carbon (C) exported to the atmosphere and coastal oceans. Climate change impacts flow regimes by increasing both flash floods and droughts. Here, we used a modelling approach to explore the consequences of lateral hydrological contraction, that is, the reduction of the wet portion of the streambed, for POM decomposition and transport at the river network scale. Our model integrates seasonal leaf litter input as generator of POM, transient storage of POM on wet and dry streambed portions with associated decomposition and ensuing changes in reactivity, and transport dynamics through a dendritic river network. Simulations showed that the amount of POM exported from the river network and its average reactivity increased with lateral hydrological contraction, due to the combination of (1) low processing of POM while stored on dry streambeds, and (2) large shunting during flashy events. The sensitivity analysis further supported that high lateral hydrological contraction leads to higher export of higher reactivity POM, regardless of transport coefficient values, average reactivity of fresh leaf litter and differences between POM reactivity under wet and dry conditions. Our study incorporates storage in dry streambed areas into the pulse-shunt concept (Raymond and others in Ecology 97(1):5–16, 2016. 10.1890/14-1684.1), providing a mechanistic framework and testable predictions about leaf litter storage, transport and decomposition in fluvial networks.

**Supplementary Information:**

The online version contains supplementary material available at 10.1007/s10021-022-00802-4.

## Highlights

- The dry streambed portion stores POM and slows down its decomposition.
- Small catchments with high flow variability export larger amounts of less degraded POM.
- Lateral hydrological contraction governs the amount and reactivity of exported POM.

## Introduction

Streams and rivers are important contributors to the global carbon (C) cycle (Cole and others 2007). The flux of organic matter (OM) that these ecosystems receive from terrestrial surroundings (that is, approximately 5.1 Pg C y^-1^) represents a sizeable proportion of the terrestrial net primary production (Aufdenkampe and others 2011). Rather than being conservatively transported to coastal areas, more than half of this OM is thought to be processed to carbon dioxide (CO_2_) and evaded to the atmosphere (Cole and others 2007; Raymond and others 2016). The processing of particulate OM (hereafter referred to as POM) by riverine biota encompassing macro- and microorganisms is a fundamental component of metabolic activity in fluvial networks (Tank and others 2010). The mineralization of POM might also play an important role for the generation of CO_2_ evading from running waters to the atmosphere, and thus, for the riverine contributions to the global C cycle. Despite continuous efforts to narrow the uncertainty of the estimates of fluvial CO_2_ evasion, how the reactivity of POM and its transport define its degradation and ultimately exports along fluvial networks remains poorly investigated. Even though this knowledge would help to mechanistically underpin the role of rivers in the global C cycle, the flux of POM along river networks is the least understood of all C species (that is, particulate organic carbon (POC), dissolved organic carbon (DOC), dissolved inorganic carbon (DIC), CO_2_ and methane (CH_4_); Tank and others (2018)).

From the many controls on POM decomposition in fluvial ecosystems (for example, chemical composition, redox conditions, solar irradiance, consumer community), hydrology is probably the most important factor as it governs exposure of POM to locally acting decomposing agents versus its withdrawal through downstream transport (Raymond and others 2016; Bastias and others 2020). Similarly to DOC, flood events shunt large amounts of POM from headwaters to downstream reaches, while increased residence times promote POM decomposition during base flow conditions (Battin and others 2008; Catalán and others 2016; Raymond and others 2016). This simple conceptual model, however, does not consider POM cycling while stored on dry portions of the streambed, which can become important in reaches where lateral hydrological contraction exposes parts of the streambed and POM thereon to the atmosphere. By *lateral hydrological contraction*, we here refer to the emergence of dry streambed fractions when flow decreases, while longitudinal flow may be maintained. The contraction implies an expansion when flow increases. This phenomenon may (or may not) be associated with intermittence, which refers to the total disappearance of superficial flow and that may co-occur gradually as longitudinal hydrological contraction at the river network scale. While the extension and implications of lateral hydrological contraction for biological and biogeochemical processes have been studied especially in intermittent and ephemeral streams, it also occurs in perennial streams across various bioclimatic regions (Steward and others 2012). Notably, flow variability and concomitant patterns of lateral hydrological contraction (and expansion) are expected to increase as a result of climate change and increased human water use (Changming and Shifeng 2002; Döll and Schmied 2012). The absence of surface water flow temporarily halts transport and limits aquatic degradation of POM, promoting its transient storage on the streambed (Larned and others 2010; Datry and others 2018; del Campo and others 2021a). Upon flow reestablishment, downstream POM transport is promoted and aquatic degradation facilitated. From a biogeochemical perspective, those alternating contraction and expansion phases have been conceptualized as a punctuated reactor (Larned and others 2010; von Schiller and others 2017). That concept states that POM may experience repeated cycles of storage (mostly on dry streambeds), transport and decomposition, that promote the degradation of POM along the river network (del Campo and others 2021b). However, the effect of lateral hydrological contraction on POM storage and degradation has not yet been evaluated at the river network scale.

Lateral hydrological contraction is expected to control storage versus transport of POM as well as the changes on its reactivity. POM here is considered to be derived from terrestrial leaf litter, which constitutes around 41% of the total mass of terrestrial plant litter inputs (Datry and others 2018), the remaining part being less reactive material such as wood. *Reactivity* is here understood as various physical and chemical features that collectively determine POM average degradation rate. As decomposition progresses and labile fractions disappear from the POM pool, overall POM reactivity decreases (that is, the distribution of decay rates shifts to lower values). The decomposition of POM is considered to be faster under wet than under dry conditions (del Campo and others 2021a). Yet, during contraction, in dry streambed fractions, physical and biological processes, for example, photodegradation and partial microbial decomposition, alter POM reactivity (that is, pre-conditioning *sensu* Abril and others 2016; Dieter and others 2011; del Campo and Gómez 2016). Preconditioning and mixing of that material with freshly fallen leaf litter has been shown to affect POM reactivity in reach-level and laboratory empirical studies (for example, del Campo and others 2021b). The relevance of those processes for the degradation and export of POM at the network scale remains poorly understood. Network-scale models can help to address this knowledge gap by integrating changes in POM reactivity as it travels from headwaters to lowlands.

Here, our objective was to explore how lateral hydrological contraction and expansion patterns influence POM decomposition and transport at the river network scale. To do so, we coupled a synthetic fluvial network model to a POM reactivity continuum model fitted to empirical degradation rates from both dry and wet streambeds. Our model captures flow regime-controlled dynamics of POM transport as well as storage and decomposition alongside modulation of the POM reactivity. We started with modelling various scenarios of lateral hydrological contraction (that is, flow variability) by controlling the frequency and intensity of effective rainfall (that is, rainfall that produces streamflow). Then, we investigated their effects on POM storage, transport and reactivity along the fluvial network. With this modelling exercise, we expand the scope of the pulse-shunt concept (Raymond and others 2016) by including the effect of POM storage on dry streambed portions and exploring its implications for POM export and decomposition in river networks experiencing different degrees of lateral hydrological contraction.

## Methods

**Figure 1:**
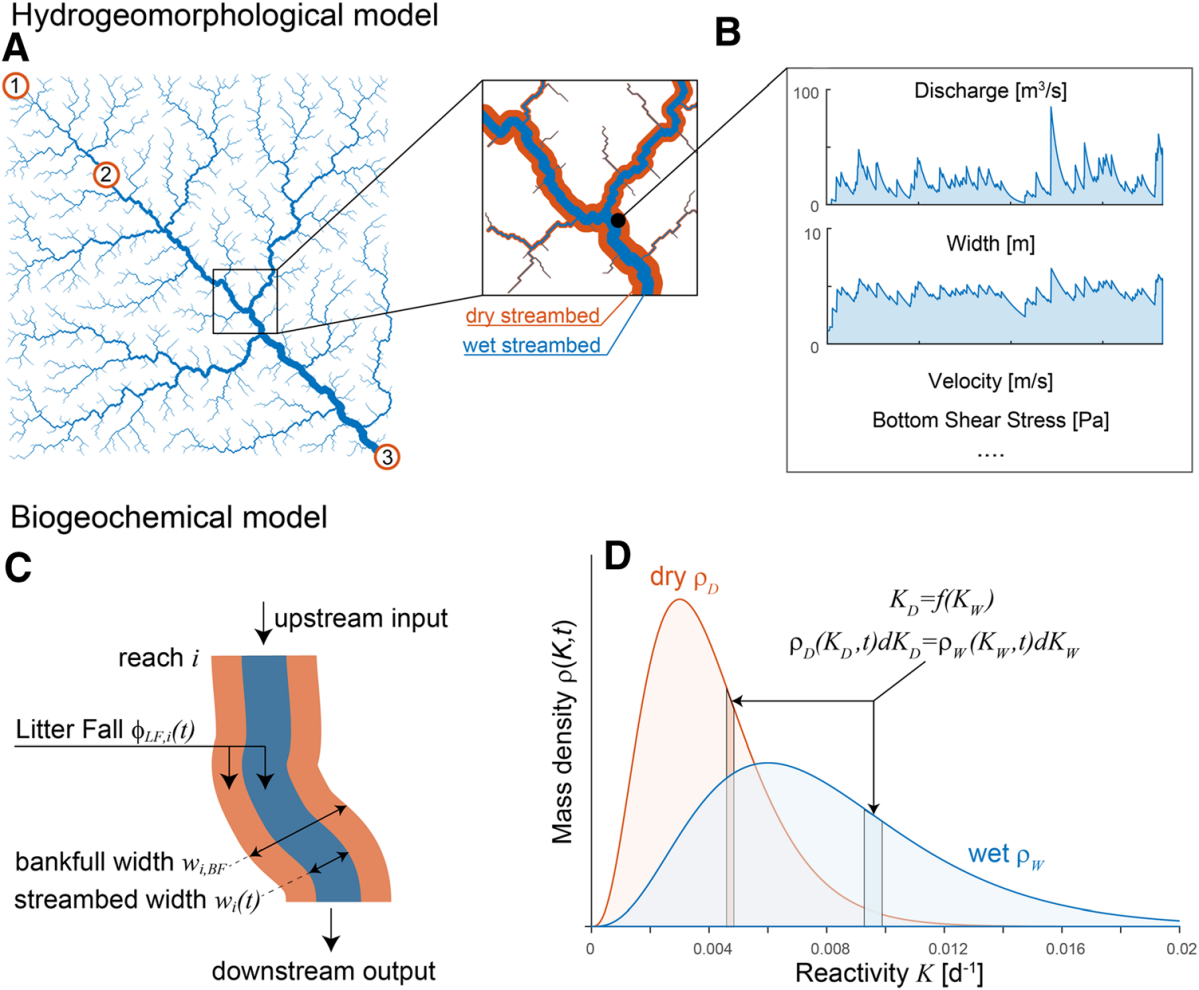
Conceptual representation of the model. **A** The river network used and highlighted the position of the outlets of three catchments whose POM dynamics will be shown in Figures 3 and 4.** B** Example of the hydrological model, used to derive stream width, depth, flow velocity and bottom shear stress. **C** Representation of the second part of the model: storage, transport and (**D**) processing of the POM

The description of the developed model consists of two main parts. In the first part, we describe how to create a synthetic river network (Figure 1A) and how to simulate realistic flow regimes with different degrees of variability. The latter goal is achieved by implementing a stochastic model of rainfall generation coupled with a hydrological model to simulate time-varying discharge in every reach of the network (Figure 1B). Through well-known hydrological scaling relations, we then derive, for each reach and at every time, the values of stream width, depth, flow velocity and bottom shear stress (Figure 1B). These hydrological variables are then exploited in the second part to model: the storage, transport and processing of POM (Figure 1C). The model accounts for four main processes: (i) POM input from seasonal litter fall, which can reach the stream or the dry streambed; (ii) exchange of POM mass between the wet and dry portions of the streambed when flow laterally contracts and expands; (iii) downstream transport of POM driven by bottom shear stress and flow velocity; and (iv) degradation of POM in both wet and dry streambed areas (Figure 1D). The latter is described through a reactivity continuum (RC) model which allowed us to track spatial–temporal variations of the average POM reactivity. The development of all model components, detailed in the following, was guided by a principle of parsimony, accounting for the most relevant processes and time scales, but limiting the number of parameters controlling the flow regime and the POM dynamics.

### Optimal Channel Networks and Hydrological Model

We simulated the spatial–temporal dynamics of POM over Optimal Channel Networks (OCNs) (Rinaldo and others 1992) obtained using the R-package OCNet (cran.r-project.org/package=OCNet). This mathematical framework generates synthetic yet realistic stream networks with the same universal fractal properties and metrics shared by all real networks (Rodríguez-Iturbe and Rinaldo 2001; Rinaldo and others 2014), and it is frequently used to generate stream network analogues for biological and ecological applications (for a review, see Carraro and others 2020). The use of OCNs gave us direct control over the size of the catchment and its drainage density (Figure 1A). Starting from a pixel description of the catchment (Carraro and others 2020), we discretized the network into stream reaches (that is, the channel segments between two consecutive confluences or between the head of a first-order stream and the next confluence) and we termed $l_i$ [L] the length of the generic reach *i* (Figure 1A). The average slope of the generic reach *i*, $s_i$ [L L^-1^] can be related to that of the catchment outlet, $s_{\text{O}}$, by the well-established scaling relation (Rodríguez-Iturbe and Rinaldo 2001): $s_i=s_{\text{O}}(A_i/A_{\text{O}})^{-0.5}$, where $A_i$ [L^2^] and $A_{\text{O}}$ represent the catchment area of reach *i* and of the outlet, respectively.

To generate synthetic time series of discharge and related hydromorphological variables in all reaches of the studied river network, we applied the following workflow. First, we generated a synthetic time series of effective (that is, streamflow producing) rainfall. Second, we modelled the hydrologic response of each subcatchment (that is, the portion of the catchment directly drained by a single stream reach) and routed the streamflow contribution of each individual subcatchment along the stream network (see details below). Finally, we employed hydraulic scaling relations (Leopold and Maddock 1953) to translate local discharge into hydraulic geometry of the channel (that is, width, depth, flow velocity, bottom shear stress) (Figure 1B).

We generated synthetic time series of effective rainfall at daily time scale events according to a simple yet realistic and widely used stochastic model (Botter and others 2007; Ceola and others 2010). The model assumes that daily events follow a marked Poisson process with rate $\lambda$ [T^-1^]: that is, every day there is a probability $\lambda \cdot \Delta t$ ($\Delta t=1$ day) that an effective rainfall event occurs. The depth of daily streamflow-producing rainfall events is a stochastic variable which follows an exponential distribution with mean equal to $\alpha$ [*L*]. We further downscaled effective rainfall from daily to hourly time scale assuming a random duration (uniformly distributed in the interval [1, 24]) and time of the beginning of the event, and that also hourly rainfall is exponentially distributed. Effective rainfall is transformed into the streamflow contribution of each subcatchment *i*, $q_i(t)$ [L^3^ T^-1^] using an exponential instantaneous unit hydrograph with average response time *u* [T]. This parameter can be thought of as the timescale of flow events after rainfall: short *u*’s imply fast, peaky catchment responses, while longer *u*’s produce a more attenuated and long-lasting hydrograph. We further assumed a baseflow discharge equal to the 0.5% of mean annual flow. This ensured that flow is always maintained and the streambed does not dry up completely. At catchment scale, we assumed the response time of channels to be much shorter than the one of hillslopes and therefore we neglected it (that is, instantaneous flood-wave propagation). Therefore, discharge at every reach *i* of the network $Q_i(t)$ [L^3^ T^-1^] can be derived as:

1 $$\begin{aligned} Q_i(t)=\sum _j W_{ji} Q_j(t) + q_i(t)\, , \end{aligned}$$

where $W_{ji}$ is the arbitrary element of the connectivity matrix $\varvec{W}$: $W_{ji}=1$ if reach *j* drains directly into *i*, and $W_{ji}=0$ otherwise. From mass balance, it follows that at any reach *i* mean discharge reads $\bar{Q}_{i}=\lambda \, \alpha \, A_i$.

Scaling relations in the downstream direction describe how channel and flow geometry change moving downstream for a discharge with a fixed frequency of occurrence (Leopold and Maddock 1953). Focusing on the mean annual flow, the channel width $\bar{w}_i$ [L] and the average depth $\bar{d}_i$ [L] of the flow in reach *i* can be approximated as

2 $$\begin{aligned} \bar{w}_{i}=B_w \bar{Q}_{i}^{\beta _w} \;\;\;\;\; \bar{d}_{i}=B_d \bar{Q}_{i}^{\beta _d}. \end{aligned}$$

The definitions of the parameters used to construct the scaling relations are provided in Table 1. To reconstruct spatial–temporal patterns of hydraulic variables during unsteady flow, equation (2) needs to be complemented with the at-a-station scaling relations, which describe how flow geometry changes with discharge at a fixed cross section (Leopold and Maddock 1953). Combining the two forms of scaling, we obtain width and depth at a given time:

3 $$\begin{aligned} w_{i}(t)=\bar{w}_i\left( \frac{Q_i(t)}{\bar{Q}_{i}}\right) ^{\alpha _{w}} \;\;\;\;\; d_{i}(t)=\bar{d}_i\left( \frac{Q_i(t)}{\bar{Q}_{i}}\right) ^{\alpha _{d}}\,. \end{aligned}$$

Note that $\bar{w}_i$ and $\bar{d}_i$ in equations (2) and (3), indicate width and depth in correspondence to the mean discharge, which differ from the mean width and depth due to the nonlinear relation between discharge and these geometric variables. Streamflow velocity ${v}_{i}$ [L T^-1^] can be derived by continuity as $v_{i}(t)=Q_i(t)/(w_{i}(t)\,d_{i}(t))$. The average bottom shear stress $\tau _i(t)$ [M L^-1^T^-2^] was used to determine POM re-suspension, which strongly controls its transport (Acuña and Tockner 2010). We estimated $\tau _i(t)$ as $\gamma \,d_i(t)\,s_i$, where $\gamma$ [M L^-2^T^-2^] is the specific weight of water. The latter equation assumes quasi-uniform flow conditions and that the hydraulic radius (that is, the area of the stream cross section divided by the wetted perimeter) is well approximated by the average flow depth. Finally, we defined the river banks as the physical boundaries of our model (that is, no interaction with the adjacent floodplain is considered; see also below). The bankfull width of a channel depends on its hydrological regime and in particular on the intensity of floods which effectively shape its geomorphological equilibrium (Leopold and others 2020). Accordingly, to estimate the bankfull width of each reach, $w_{\text{BF},\,i}$, we ran a 200 years long simulation of the hydrological model and we approximate $w_{\text{BF},\,i}$ as the channel width corresponding to the discharge with a recurrence interval of 2 years (Wilkerson 2008; Leopold and others 2020).

**Table 1:** Parametrization for the Hydrological and POM Degradation Components of the Model

Symbol	Definition	Units	Value	Source
$s_{\text{O}}$	Average slope of the outlet reach	–	0.001	–
$B_w$	Parameter of downstream scaling relation $\bar{w}_{i}=B_w \bar{Q}_{i}^{\beta _w}$	m^(1-3βw)^ s^βw^	10	Leopold and Maddock (1953)
$\beta _w$	Exponent of downstream scaling relation $\bar{w}_{i}=B_w \bar{Q}_{i}^{\beta _w}$	–	0.5	Leopold and Maddock (1953)
$B_d$	Parameter of downstream scaling relation $\bar{d}_{i}=B_d \bar{Q}_{i}^{\beta _d}$	m^(1-3βd)^ s^βd^	0.25	Leopold and Maddock (1953)
$\beta _d$	Exponent of downstream scaling relation $\bar{d}_{i}=B_d \bar{Q}_{i}^{\beta _d}$	–	0.4	Leopold and Maddock (1953)
$\alpha _w$	Exponent of the at-a-station scaling relation $w_{i}(t)\propto Q _i(t)^{\alpha _{w}}$	–	0.26	Leopold and Maddock (1953)
$\alpha _d$	Exponent of the at-a-station scaling relation $d_{i}(t)\propto Q_i(t)^{\alpha _{d}}$	–	0.4	Leopold and Maddock (1953)
$\langle K_{\text{W, LF}} \rangle$	Average reactivity of leaf litter in wet conditions	d^-1^	0.005, **0.01**, 0.02	Supporting Information
$\nu$	Shape factor of the $p_{\text{W}}$ gamma distribution	–	1	Supporting Information
$F_{\text{WD}}$	Ratio between dry and wet reactivities ($K_{\text{D}}/K_{\text{W}}$)	–	0.1, **0.2**, 0.3	Supporting Information
$\tau _0$	Bottom shear stress to initiate POM transport	Pa	8	“Parameters and Sensitivity Analysis” section
$\mu$	Transport coefficient	10^-3^ Pa^-1^	12, **30**, 46	“Parameters and Sensitivity Analysis” section

When three values are reported, they refer to the range investigated in the sensitivity analysis (bold fonts indicate the reference value used to obtain the results shown in Figures 3 and 4).

### Leaf Litter Transport and Degradation Model

To capture the changes in physical and chemical properties of leaf litter controlling its degradation rates, we used the reactivity continuum (RC) theory (Boudreau and Ruddick 1991). This representation can be thought of as a particular case of the more general q-theory (Bosatta and Ågren 1985) and considers a continuous spectrum of reactive types within OM, instead of a discrete set of pools of OM with contrasting decay rates (Arndt and others 2013). In this context, the reactivity of a certain compound (that is, conceptually its “likeliness to be decomposed”) is identified by the associated first-order decay rate *K* [T^-1^] and directly translates to a differentiation of OM through chemical and/or physical state. Operationally, POM mass is described via the mass density $\rho (K,t)$, where $\rho (K,t){\text{d}}K$ represents the infinitesimal mass of reactivity around *K* at time *t*. Considering that each component comprised in the POM mixture decays following a first-order kinetic according to its reactivity *K*, the density $\rho (K,t)$ changes in time following the ordinary differential equation:

4 $$\begin{aligned} \frac{{\text{d}} \rho (K,t)}{{\text{d}}t}=-K\rho (K,t) \end{aligned}$$

As the degradation process progresses, the more labile components (high *K*) decay more rapidly and therefore the average reactivity of POM decreases (Figure 1D).

At any time *t*, depending on discharge, a fraction $w_i(t)/w_{\text{BF},\,i}$ of the streambed of reach *i* is wet while the remaining fraction is assumed to be dry (that is, no flowing water). The total mass of leaf litter can likewise be apportioned between the wet and dry fractions (Figure 1C). Therefore, following the RC theory introduced above, the state variables describing POM in a stream reach are $\rho _{{\text{D}},\,i}(K_{\text{D}},t)$ and $\rho _{{\text{W}},\,i}(K_{\text{W}},t)$ [M T], which quantify the density of POM mass of different reactivities in, respectively, the dry and wet streambed portion of reach *i* at time *t* (Figure 1D). The POM mass in the dry fraction of the streambed of reach *i* thus reads:

5 $$\begin{aligned} m_{{\text{D}},\,i}(t)=\int _0^\infty \rho _{{\text{D}},\,i}(K_{\text{D}},t) \,{\text{d}}K_{\text{D}}\,. \end{aligned}$$

The corresponding wet variables $\rho _{{\text{W}},\,i}(K_{\text{W}},t)$ and $m_{{\text{W}},\,i}(t)$ are defined analogously. In addition, we assume a characteristic relationship between dry $K_{\text{D}}$, and wet $K_{\text{W}}$ degradation rates, so that a given POM with a degradation rate $K_{\text{W}}$ when exposed to wet streambed conditions, will experience a slower degradation rate $K_{\text{D}}=f(K_{\text{W}})$ when transferred to a dry environment. When a given pool of POM with a range of different reactivities goes from wet to dry conditions or vice versa, mass conservation implies the following relationship between the two mass densities (Figure 1D):

6 $$\begin{aligned} \rho _{\text{W}}(K_{\text{W}},t){\text{d}}K_{\text{W}}&=\rho _{\text{D}} (K_{\text{D}},t){\text{d}}K_{\text{D}}=\rho _{\text{D}} (f(K_{\text{W}}),t)\text{d}K_{\text{D}} \rightarrow   \\ \rho _{\text{W}}(K_{\text{W}},t)&=\rho _{\text{D}}(f(K_{\text{W}}),t)\frac{\text{d}K_{\text{D}}}{\text{d}K_{\text{W}}}=\rho _{\text{D}}(f(K_{\text{W}}),t)\frac{\text{d}f(K_{\text{W}})}{\text{d}K_{\text{W}}}\,. \end{aligned}$$

For any reach *i* of the network, the four processes illustrated above (that is, leaf litter input, exchange of POM between wet and dry streambed areas, transport and degradation) are described by the following partial differential equations for the POM mass densities:

7 $$\begin{aligned} \frac{\partial }{\partial t}\rho _{{\text{D}},\,i}(K_{\text{D}},t)&=\phi _{\text{LF}}(t)\,l_i\, p_{\text{D}}(K_{\text{D}}) \left( 1-\frac{w_i(t)}{w_{\text{BF},\,i}}\right) -K_{\text{D}}\,\rho _{{\text{D}},\, i}(K_{\text{D}},t) - F_{i, {\text{D}} \rightarrow {\text{W}}}(t) \end{aligned}$$

8 $$\begin{aligned} \frac{\partial }{\partial t}\rho _{{\text{W}},\,i}(K_{\text{W}},t)&=\phi _{\text{LF}}(t)\,l_i\, p_{\text{W}}(K_{\text{W}}) \frac{w_i(t)}{w_{\text{BF},\,i}}-K_{\text{W}}\; \rho _{{\text{W}},\,i}(K_{\text{W}},t) + F_{i, {\text{D}} \rightarrow {\text{W}}}(t)  \\ &\quad + \sum _{j}W_{ji}\delta _j(t)\rho _{{\text{W}},\,j}(K_{\text{W}},t)- \delta _i(t)\rho _{{\text{W}},\,i}(K_{\text{W}},t)\, , \end{aligned}$$

where $\phi _{\text{LF}}(t)$ [M L^-1^ T^-1^] is the input of POM (as leaf litter) from the terrestrial ecosystem per unit of time and of stream length. At any time, this input is partitioned into the wet and dry streambed according to the concurred stream width $w_i(t)$. $p_{\text{D}}(K_{\text{D}})$ and $p_{\text{W}}(K_{\text{W}})$ [T] are the distributions of reactivity of the fresh POM derived from litter fall under dry and wet conditions, respectively. The relation between the two distributions follows the same rationale proposed in equation (6): $p_{\text{W}}(K_{\text{W}})=p_{\text{D}}(f(K_{\text{W}}))\text{d}f/\text{d}K_{\text{W}}$. The second terms of the right hand side of equations (7) and (8) represent, in analogy to equation (4), the first-order decay of the different POM constituents. When a patch of streambed dries out (or is submerged) due to hydrological contraction (or expansion), the corresponding POM mass is transferred to the dry (or wet) counterpart. The flux $F_{i, {\text{D}} \rightarrow {\text{W}}}(t)$ quantifies such exchange. Specifically, $F_{i, {\text{D}} \rightarrow {\text{W}}}(t)$ [M T T^-1^] is the density mass flux from the dry to the wet fraction and it is thus positive when flow is increasing and negative otherwise. Assuming a uniform distribution of areal POM concentration within each reach, this flux reads:

9 $$\begin{aligned} F_{i, {\text{D}} \rightarrow {\text{W}}}(t) = {\left\{ \begin{array}{ll} \frac{{\text{d}}w_i(t)}{{\text{d}}t} \frac{\rho _{{\text{D}},\,i}(K_{\text{D}},\,t)}{ w_{\text{BF},\,i}-w_i(t)}  \,\,{\text{if}}\,&{} \frac{{\text{d}}w_i(t)}{{\text{d}}t} > 0 \\ \frac{{\text{d}}w_i(t)}{{\text{d}}t} \frac{\rho _{{\text{W}},\,i}(K_{\text{W}},\,t)}{w_i(t)} \,\,{\text{if}}\, &{} \frac{{\text{d}}w_i(t)}{{\text{d}}t} \le 0 \end{array}\right. } \end{aligned}$$

It should be noted that when $F_{i, {\text{D}} \rightarrow {\text{W}}}(t)$ represents an input (that is, increasing flow for $\rho _{{\text{W}},\,i}$ and decreasing flow for $\rho _{{\text{D}},\,i}$), the mass density must be transformed following equation (6) to account for the change in reactivity of the same material in the two contrasting environments. The last two terms of the right hand side of equation (8) simulate the downstream transport of POM which is assumed to occur at the site-specific and time-varying rate $\delta _i(t)$. The input in reach *i* consists of the sum of the downstream transport of the immediately upstream reaches. Transport of POM is a complex phenomenon that involves the continuous processes of re-suspension, transport in the water column and sedimentation and settlement of particles. Here, we adopted a simplified approach, inspired by Acuña and Tockner (2010), aimed at modelling the bulk rate at which POM moves downstream. The concentration of suspended POM depends on the balance between re-suspension and sedimentation rates. While the former depends on the particle size distribution, the latter crucially depends on hydraulic conditions, specifically on the bottom shear stress $\tau _i(t)$, which, when above a critical threshold $\tau _0$ that initiates particles movement, promotes re-suspension. The suspended concentration is thus considered to be proportional to the excess of shear stress $(\tau _i(t)-\tau _0)$. Assuming that suspended particles are advected by the flow velocity $v_i(t)$, the bulk velocity at which POM move downstream is proportional to the product between shear stress excess and flow velocity: $(\tau _i(t)-\tau _0) v_i(t)$. Finally, the rate $\delta _i(t)$ at which POM leaves reach *i* can be computed dividing the bulk velocity by the reach length $l_i$:

10 $$\begin{aligned} \delta _i(t)=\mu \cdot (\tau _i(t)-\tau _0)H(\tau _i(t)-\tau _0)\,\frac{v_i(t)}{l_i} \end{aligned}$$

where $\mu$ is a model parameter and $H(\cdot )$ is the Heaviside step function which is equal to 1 when $\tau _i>\tau _0$ and 0 otherwise.

The model described above aims at simulating the relevant processes within river banks. During large floods, discharge may exceed the bankfull discharge with the flow inundating the adjacent floodplain. According to our assumptions, such events occur on average every 2 years (see “Optimal Channel Networks and Hydrological Model” section). Overbank flow could promote an exchange of POM between the stream and the riparian ecosystems, which, however, has been neglected here because it is beyond the scope of the present investigation. Mathematically, when $Q_i>Q_{\text{BF},\, i}$, we assumed $\rho _{{\text{D}},\,i}=0$ and do not model any further input nor exchange of POM mass between wet and dry streambed fractions.

### Model Set Up

We started from an OCN network derived from a $512\times 512$ pixel square domain. We assumed each pixel to have a 100-meter-long edge, thus leading to a total catchment area of 2625 km^2^. We extracted the channel network using a threshold of 1 km^2^ (Figure 1A) which resulted in 1465 stream reaches with a total length of 1839 km. Parameters defining the channel and flow geometry are reported in Table 1 along with the corresponding literature references. We generated the scenarios of lateral hydrological contraction based on three levels of flow variability, obtained by changing the coefficient of variation of the streamflow (CV(*Q*)). To do so, we varied the frequency of occurrence of rainfall events $\lambda$ and the catchment response time of the hydrograph *u* as reported in Table 2. The effective rainfall mean depth $\alpha$ is computed so that all scenarios share the same average annual effective precipitation depth of 250 mm. Thus, the three scenarios share also the same average annual flow. This assumption allowed us to investigate the effect of lateral hydrological contraction caused by flow variability separately from that possibly induced by different mean flows. Thus, the scenario with high degree of lateral contraction emerges from a high coefficient of variation of the stream width (CV(*w*), Table 2), resulting from high streamflow variability (that is, a flashy flow regime characterized by few large flow events with a fast response time). On the other hand, the scenario of low lateral hydrological contraction has a low degree of flow variability (that is, frequent flow events with a slow response time). Note that we do not aim to capture the flow regime of an intermittent river but the spatial–temporal dynamics of lateral hydrological contraction.

**Table 2:** Manipulation of Flow Variability Creates Three Lateral Hydrological Contraction Scenarios: Low, Mid and High

Lateral hydrological contraction scenario	$\lambda$ [d^-1^]	*u* [d]	CV(*Q*)	CV(*w*)	Total streambed area [km^2^]	Average wet streambed area [km^2^]
Low	0.5	20	0.32	0.07	9.82	8.47
Mid	0.2	10	0.68	0.17	11.75	8.22
High	0.05	5	1.93	0.58	16.57	6.10

$\lambda$ is the frequency of occurrence of rain event and *u* the response time of the catchment hydrograph. CV(*Q*) and CV(*w*) are the coefficients of variation of streamflow *Q*(*t*) and stream width *w*(*t*). The total and the average wet streambed areas are also shown. Note that by construction, all reaches have the same CV(*Q*) and CV(*w*).

We assumed an annual leaf litter input per unit length of 2.4 kg m^-1^y^-1^, according to values for POM mass reported in previous literature (Acuña and others 2007). However, note that this value does not affect model results that are presented as normalized values with respect to the total leaf litter input (for example, the fraction exported or degraded). To capture the seasonality of leaf litter input with most input flux concentrated in autumn, we assume that its annual time series, $\phi _{\text{LF}}(t)$, follows a Gaussian distribution with the mean being the Julian day 300 and a standard deviation of 20 d.

Equations (7) and (8) are integrated numerically using a forward Euler scheme with an adaptive timestep (longer allowed timestep: $\Delta t = 1$ h). The model was implemented in MATLAB R2019b. As the time series of streamflow, and of all associated hydraulic variables, is stochastic, we ran 10 years long simulations to achieve statistically representative results. Each simulation is preceded by a spin-up period of 2 years to lose memory of the initial conditions, which are null for both $\rho _{{\text{D}},\,i}$ and $\rho _{{\text{W}},\,i}$. For each hydrological contraction scenario, we quantified POM export, and its degradation and average reactivity in dry and wet conditions. We did so for a series of 72 catchments with progressively larger area whose outlet belong to the network backbone (Figure 1A). These catchments expand from headwaters (point 1 in Figure 1A, catchment area: 2 km^2^) to intermediate catchments (point 2, 148 km^2^), up to the network’s outlet (point 3, 2621 km^2^). This exercise allowed us to explore changes in the fate of POM along the river continuum. The average reactivity $\langle K_{\text{W}} \rangle$ of the POM exported from the network was calculated as:

11 $$\begin{aligned} \langle K_{\text{W},\,i} \rangle (t)=\frac{\int _0^\infty \rho _{{\text{W}},\,i}(K_{\text{W}},t)K_{\text{W}} \,\text{d}K_{\text{W}}}{m_{W,\,i}(t)}\,. \end{aligned}$$

The upper bound of $\langle K_{\text{W},\,i} \rangle$ is the average reactivity of the fresh leaf litter: $\langle K_{\text{W},\,\text{LF}} \rangle$, i.e. the average of the distribution $p_{\text{W}}(K_{\text{W}})$ in equation (8). When leaf litter is processed, the more labile components are degraded at a faster rate and the relative abundance of the more recalcitrant materials increases, and thus the average reactivity decreases.

### Parameters and Sensitivity Analysis

We performed model simulations and assessed the influence of lateral hydrological contraction of POM dynamics based on a reference set of model parameters (Table 1) estimated from literature values and expert knowledge (see below). As some parameters are particularly uncertain and potentially different depending on POM composition and environmental conditions, we further ran a sensitivity analysis varying the values of some selected parameters around the reference ones. The goal of this analysis was to check whether patterns of POM fate and average reactivity found for the reference set are robust to variations of such parameters. The sensitivity analysis was repeated for the low, mid and high lateral hydrological contraction scenarios as defined in Table 2.

To estimate parameters related to the leaf litter reactivity distribution, we collected information on degradation of POM from available literature (Supplementary Table S1). Usually, in these experiments, leaves from a given tree species are placed inside a mesh bag and left in or on the streambed for a period of time to monitor their mass loss, measured as dry mass or ash free dry mass (AFDM). Typically considered habitats represent the aquatic-terrestrial habitat mosaic known for intermittent rivers: remnant pools, gravel bars, shores and channel sediments, and flowing water. Two types of experiments were represented in the selected database: i) sequential: when the bags were exposed first to wet and then to dry conditions, or vice versa; and ii) those in which bags with the same material were exposed to wet and dry conditions simultaneously. Here, as in the reviewed studies, we considered AFDM as a good proxy for POM in the model. We extracted literature information on time (days) of bag collection, corresponding AFDM loss, habitat and dry or wet conditions from a total of n=19 time series (Supplementary Information). We assumed the initial distribution of leaf litter reactivity (say, in wet conditions: $p_{\text{W}}(K_{\text{W}})$) to follow a Gamma distribution which proved to be a flexible and effective descriptor of OM reactivity (Koehler and Tranvik 2015; Catalán and others 2017). Such a distribution is controlled by two parameters: the rate $\kappa$ [T] and the shape $\nu$ [−]. The average reactivity of leaf litter $\langle K_{\text{LF}} \rangle$ is equal to $\nu /\kappa$ and represents the initial apparent first-order decay rate. We assumed a simple linear function to describe the change in reactivity from wet to dry conditions: $K_{\text{D}}=f(K_{\text{W}})=F_{\text{WD}}\,K_{\text{W}}$, where the parameter $F_{\text{WD}}$ can be thought of as the ratio between the dry and wet degradation rates of a specific material. We fitted the RC degradation model to the experimental time series using the transformation in equation (6) to simulate the change in degradation kinetics between the two contrasting environments. For every time series, we jointly estimated the three model parameters ($\nu$, $\langle K_{\text{W},\text{LF}} \rangle$ and $F_{\text{WD}}$) minimizing the root mean square error between simulated and observed AFDM time series (Figures S1 and S2). When starting from a Gamma distribution, the degradation of the total mass can be described analytically as $\text{AFDM}(t) =\text{AFDM}(t\!=\!0) \cdot (\kappa /(\kappa +t))^{\nu }$ (Boudreau and Ruddick 1991). This solution can be applied to the non-sequential experiments and to the first phase of the sequential ones. For the second phase of the sequential experiments, the analytical solution cannot be applied because the initial reactivity distribution is not a gamma distribution. We then integrated the RC model numerically in these cases. Results are summarized in Table S1 and Figure S3. For our modelling experiments, we chose a value $\nu =1$, which is representative of the median experimental value. For the sensitivity analysis, we explored three values of the parameters $\langle K_{\text{W},\text{LF}} \rangle$ and $F_{\text{WD}}$ that covered the bulk of the experimental distribution (see Table 1). Specifically, we varied the average reactivity of leaf litter in wet conditions ($\langle K_{\text{W},\text{LF}} \rangle$), which determines initial POM reactivity, from 0.005 to 0.02 d^-1^ (reference value: 0.01 d^-1^). The ratio between dry and wet reactivity ($K_{\text{D}}/K_{\text{W}}$) was changed from 0.1 to 0.3 (reference value: 0.2).

The simulation of POM transport was controlled by two variables $\tau _0$, the average bottom shear stress above which transport occurs, and $\mu$, the transport coefficient. We ensured $\tau >\tau _0$ at least 25% of the time in all reaches and for all the three flow regimes by manually adjusting the value of $\tau _0$ to 8 Pa (Table 1). This procedure allowed the analysis of scenarios where both degradation and transport of POM occur simultaneously, avoiding bottlenecks that would prevent the simulation of POM transport downstream. The coefficient $\mu$ controls the rate at which POM is transported downstream. This parameter is difficult to estimate or to infer from the literature as it arguably depends on the size distribution of POM particles and on the streambed grain-size distribution. We selected $\mu =0.03$ Pa^-1^ as a reference value of this parameter, which led to simulated conditions where both POM export and degradation occurred and allowed us to estimate the effect of lateral hydrological contraction on POM dynamics. To test if the resulting pattern was a genuine effect of hydrological contraction independent of the chosen value of $\mu$, we explored in the sensitivity analysis three different values covering a wide range of transport rates (Table 1).

## Results

**Figure 2:**
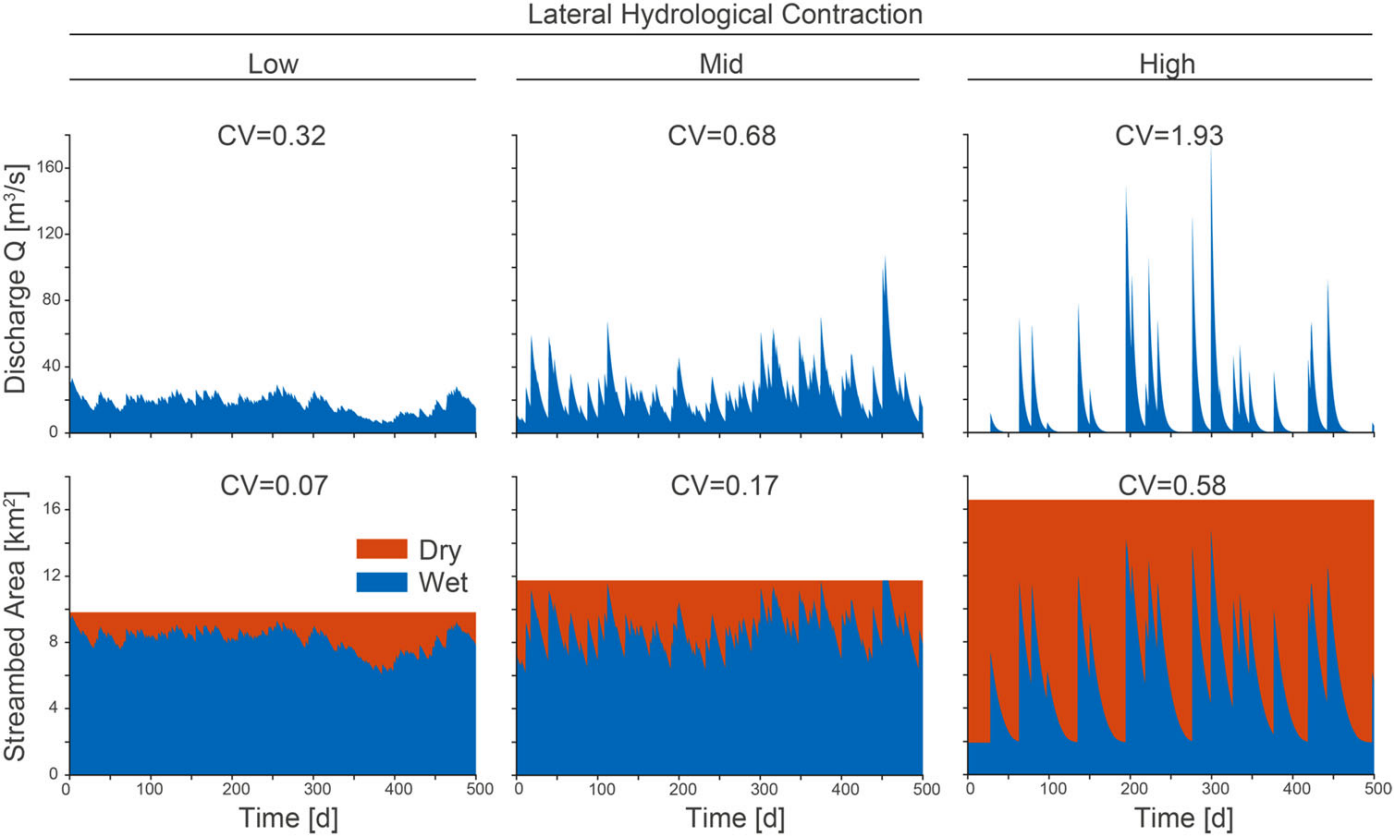
Three levels of flow variability drive scenarios of low, mid and high lateral contraction in a period of 500 days. *Top row* shows the temporal variability of discharge at the outlet of the river network. *Bottom row* shows the temporal variability of total, wet and dry streambed areas. Coefficients of variation (CV) of both discharge and wet streambed area are also reported. Note that by construction, CV of wet streambed area and flow width (CV(*w*), Table 2) are the same.

The model generated three contrasting lateral hydrological contraction scenarios as a result of modifying flow variability (Figure 2). Low flow variability (see coefficients of variation in Table 2) led to smaller total streambed area with little lateral contraction. As flow variability increases, in the mid and high scenarios, so does the total streambed area, linked to events with higher discharge and thus larger bankfull width and larger dry streambed areas during contraction periods.

**Figure 3:**
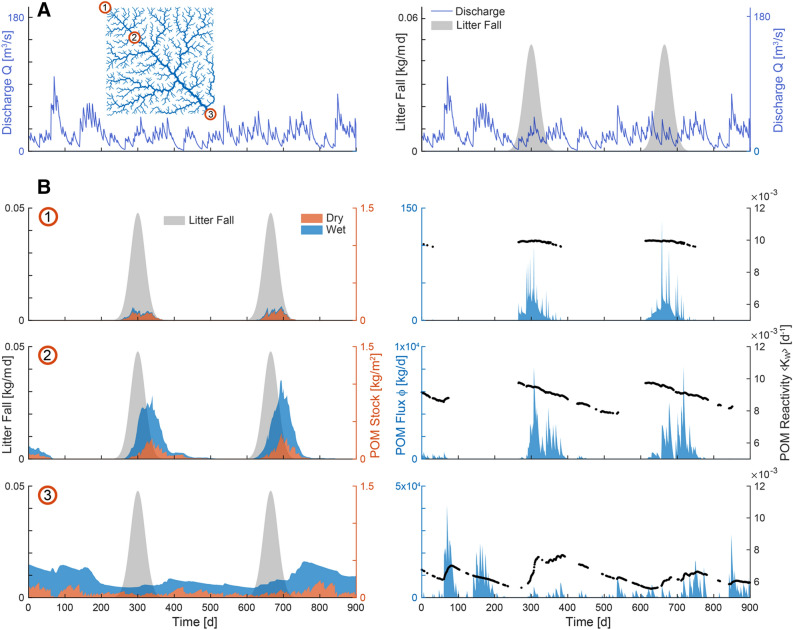
Example of model simulation for mid lateral hydrological contraction scenario and reference parameter set (Table 1) for a time window of 900 days. **A** Discharge at the outlet and time series of litter fall (*only right panel*) to ease the readability of the results reported in (**B**). **B** POM stock, flux and reactivity in time. Stock indicates the local POM mass accumulated on the dry and wet streambed fractions of the selected reach. POM flux refers to the POM exported downstream (only in wet conditions with sufficient bottom shear stress) and POM reactivity to the average reactivity of that exported POM (equation 11). Numbers from 1 to 3 indicate three different stream reaches that are outlets of three corresponding catchments with increasing size (see also Figure 1). Analogous figures for the low and high flow variability scenarios are reported in Figures S4 and S5, respectively.

There was a distinct spatial–temporal pattern in POM storage along the river continuum, which is shown for the mid lateral contraction scenario using the reference parameter set (see Table 1) in Figure 3. In small headwater reaches (for example, site 1 in Figure 3B), POM accumulated on the streambed only during the leaf fall period and was rapidly shunted downstream. The steep slopes and the ensuing high bottom shear stress in small headwaters favoured POM transport rather than storage, which mostly occurred on the dry streambed. The partition of POM stocks between the dry and wet fractions of the streambed changed in reaches draining mid-size catchments (site 2 in Figure 3B), where input from upstream favoured the presence and degradation of POM on the wet streambed. A non-negligible stock of POM was stored in the dry fraction through the winter and into the following spring. Finally, for the largest catchment, represented at the network outlet (site 3 in Figure 3B), POM stocks occurred both on the wet and dry streambed fractions year-round and with an attenuated seasonality. From the small-size catchments to the outlet, the peak in POM storage was progressively delayed with respect to leaf fall because the overall residence time of POM increased with catchment area (Figure 3). The dynamics of POM flux being transported, resulted from the interaction between stocks and flow events capable of transporting POM (Figure 3B). The spatial–temporal pattern of POM storage and transport determined the variability of average reactivity of the transported POM (Figure 3B). The average reactivity of POM exported from small headwater reaches was relatively high and close to that of the fresh leaf litter ($K_{\text{W},\,\text{LF}}$= 0.01 d^-1^) because the material was quickly shunted downstream. The same occurred in reaches located in the mid-sized catchment at the beginning of the leaf fall season but, later on, average reactivity declined due to the degradation of the most labile components and the input of processed POM from upstream reaches. At the outlet of the catchment, POM reactivity also tended to be higher after the leaf fall season, though values were generally lower compared to small and mid-size catchments because of continuous presence of old and more recalcitrant POM persisting from the previous season. Interestingly, large flow events can deliver POM into a downstream reach that is fresher than the material accumulated there, with a resulting increase of the average reactivity (see, for example, around day 100 for site 3, Figure 3B). Results on POM storage, export and reactivity along the fluvial network hold for both the high and low flow variability scenarios (Supporting Figures S4 and S5). However, as lateral hydrological contraction increases, the coupling between leaf litter input and POM storage becomes weaker and there is substantial storage of POM on the dry fraction of the streambed throughout the whole hydrological year.

**Figure 4:**
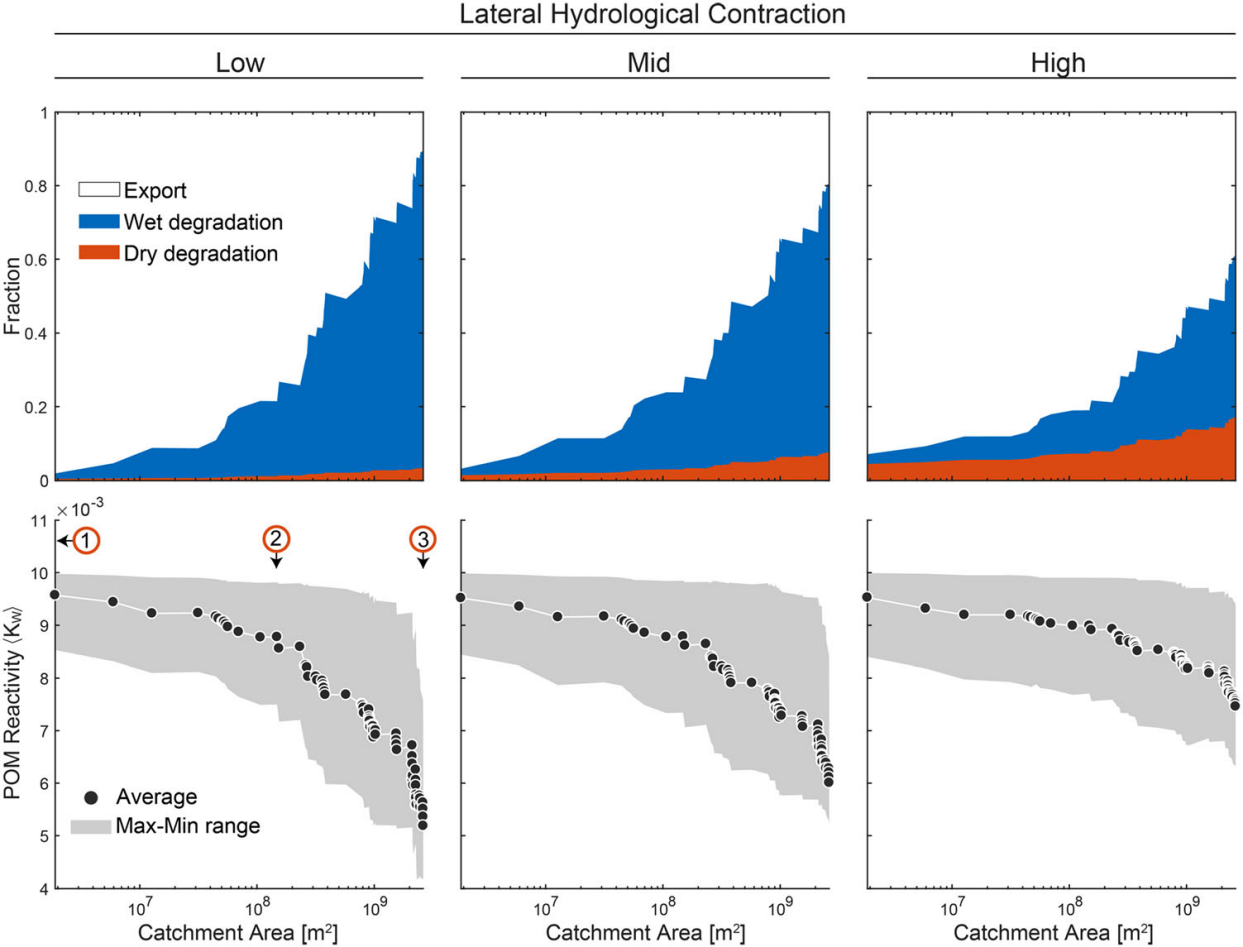
Proportion of POM subject to the three possible fates: export, wet degradation and dry degradation (*upper panels*); and reactivity of the exported POM (*lower panels*) as a function of catchment area for the three scenarios of hydrological contraction. Values are computed at the stream network scale: Each point in the *x*-axis represents a modelled subcatchment along the main backbone of the river network. Given the total leaf litter input that entered the stream network upstream of the selected point, *top panels* show the proportion undergoing the three possible fates. POM reactivity was calculated at the outlet of each of those subcatchments. Numbers 1, 2 and 3 are given as a reference and correspond to position within the network as shown in Figures 1 and 3.

The fate of POM at the stream network scale (i.e. export or degradation) was a function of catchment area and hydrological contraction (Figure 4). The larger the catchment, the lower the proportion from total POM inputs exported downstream, but the higher the proportion subjected to degradation on either wet or dry streambed portions. Lateral hydrological contraction interacted strongly with this pattern, with higher contraction leading to a higher proportion of export and a higher proportion of dry degradation. The average and distribution of reactivity of the exported POM was also a function of catchment area and hydrological contraction (Figure 4). POM reactivity decreased with catchment area, but this decrease was less pronounced as hydrological contraction increased. This pattern is due to the higher proportion of POM stored on the dry streambed in the high contraction scenario (Figure S5) and the lower exchange of POM between wet and dry portions of the streambed. Since degradation rates are lower on the dry streambed, the exported POM will be less degraded (i.e. more reactive). The downstream transport (i.e. export) of more reactive POM is facilitated by high peak flow events occurring in the high flow variability scenario with higher contraction.

**Figure 5:**
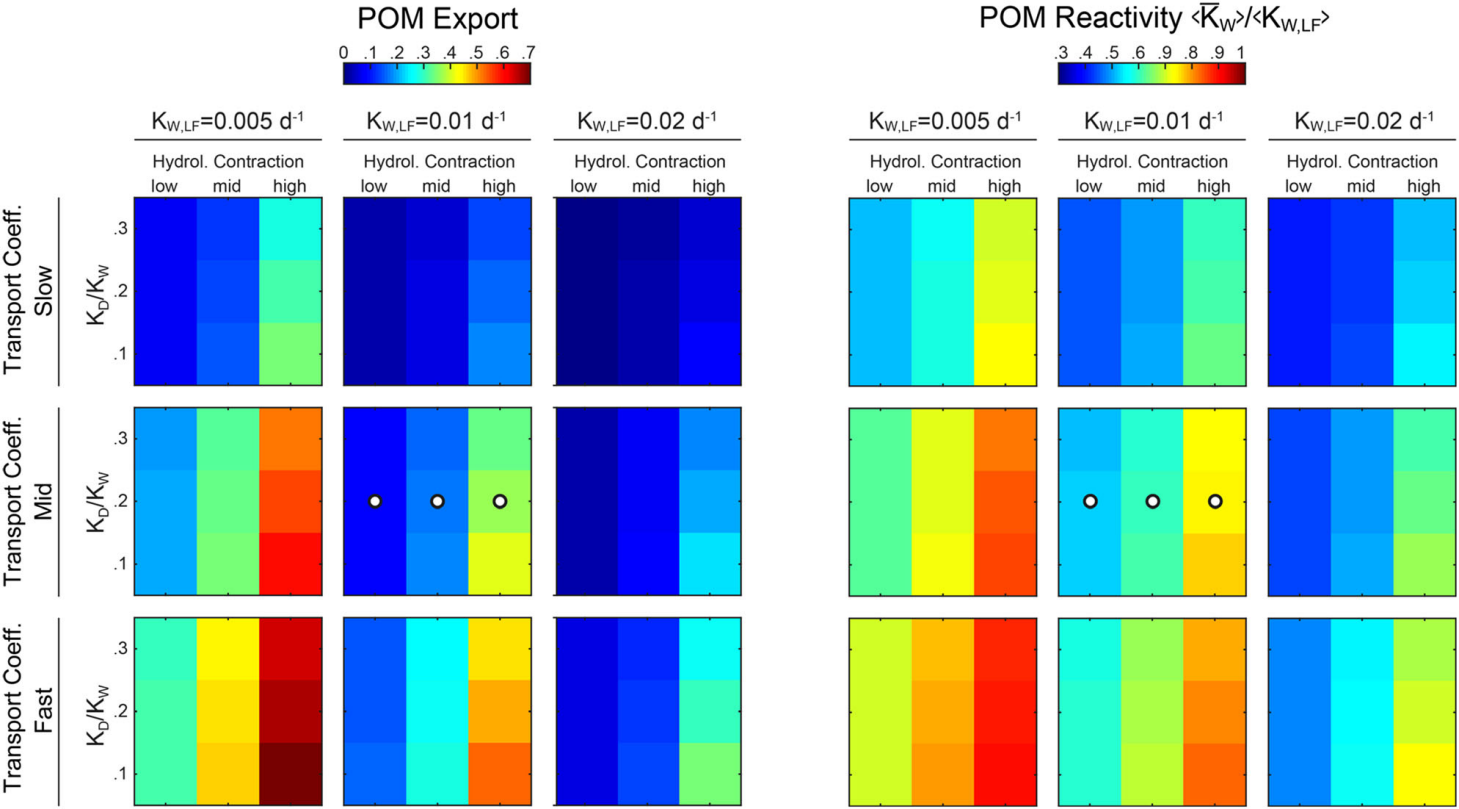
Analysis of fraction of POM exported at the outlet of the stream network (*left*) and its average reactivity (*right*; normalized with respect to the reactivity of the fresh leaf litter ($K_{\text{W}}/K_{\text{W},\,\text{LF}}$)) for different scenarios of: transport coefficient ($\mu$), average reactivity of the leaf litter input, $\langle K_{\text{W},\,\text{LF}} \rangle$, ratio between dry and wet degradation rate, ($F_{\text{WD}}=K_{\text{D}}/K_{\text{W}}$), (see values in Table 1) and hydrological contraction (see Table 2). *White dots* show the reference parameter set.

The sensitivity analysis shows that the effect of hydrological contraction on the flux of exported POM and its reactivity holds, regardless of the specific values considered for the three explored parameters (Figure 5). Indeed, for any tested combination of transport coefficient ($\mu$), average reactivity of the fresh leaf litter ($K_{\text{W},\text{LF}}$) and ratio of dry versus wet reactivity ($K_{\text{D}}/K_{\text{W}}$), the high lateral hydrological contraction scenario was the one leading to the higher proportion of POM exported with higher reactivity. Moreover, the effect of initial POM reactivity, transport and dry versus wet reactivity on POM export and final reactivity followed the expected pattern according to our model conceptualization. First, higher initial reactivity of POM decreased its downstream export, as it is more likely for it to be degraded within the river network. Second, faster transport leads to higher export of less degraded material (i.e. with $K_{\text{W}}$ more similar to that of fresh leaf litter), because the residence time of POM within the river network decreases, thus decreasing the opportunity of being degraded. Third, the higher the ratio between dry and wet reactivity, the less POM is exported and with lower reactivity as the chance of being degraded on the dry streambed portion increases.

The results from the sensitivity analysis show that the quantity of exported POM was maximized under relatively fast transport, low initial POM reactivity and low ratio of dry versus wet reactivity. This combination of parameters was also the one leading to the minimal change in POM reactivity, with the reactivity of the exported POM being similar to fresh leaf litter ($K_{\text{W},\text{LF}}$). The opposite pattern was found (i.e. minimal export of POM) under relatively slow transport, high initial POM reactivity and high ratio of dry versus wet reactivity. This pattern was accentuated for the low lateral hydrological contraction scenario. Moreover, this combination of parameters led to the highest decrease in POM reactivity compared to initial conditions.

## Discussion

In our model, flow variability was used to generate lateral hydrological contraction, which is characteristic of any river network but might affect more extensively, for example, intermittent river networks. The degree of lateral hydrological contraction determined the amount and reactivity of terrestrial POM being exported downstream, with increasing contraction resulting in enhanced export of less processed POM at the river network outlet. Discharge dynamics drove the magnitude and spatial–temporal pattern of wet width and also shear stress which together controlled transport and storage of POM along the fluvial network. Our model explicitly included the dry portion of the streambed as a relevant biogeochemical component of the river network, which has characteristic degradation rates and changes in size over space and time as a function of the hydrological regime. Thus, our model allows exploration of how the variability of the flow regime determines the amount and reactivity of the terrestrial POM stored and processed on either dry or wet streambed portions and ultimately exported to the outlet of the fluvial network.

### Model Representation of Lateral Hydrological Contraction and Dry Streambed Fractions

Our model captures the lateral hydrological contraction and expansion along the fluvial network linked to streamflow variability. Higher flow variability results in more intense floods during expansion and a larger dry streambed fraction during contraction. The higher lateral hydrological contraction scenario (Figure 2) is characterized by fast and peaky hydrographs similar to those reported for intermittent streams (see, for example, Fovet and others 2021; Sauquet and others 2021), while the low and mid hydrological contraction scenarios are more representative of hydrographs observed in streams with low or no flow intermittence. When compared with the estimated fraction of dry and wet streambed at the global scale (Messager and others 2021), the low hydrological contraction scenario leads to similar percentages (average 14%) as reported for cool temperate and moist climates, whereas the percentages for the high hydrological contraction scenario (average 63%) are similar to those measured in cool and xeric regions. Therefore, the simulated hydrological conditions were representative of a large part of the global river network, as perennial streams also experience some degree of lateral contraction (Uys and O’keeffe 1997). Moreover, peaky hydrographs (including both extreme precipitation and no-flow days) are increasingly linked to climate change in multiple regions (Sauquet and others 2021). As we exploit mechanistic understanding of leaf litter inputs, POM transport and reactivity distribution changes, our effort is a suitable approach developed in a synthetic catchment, to reliably model effects of lateral hydrological contraction on POM processing and export.

We can, for instance, test some of the predictions made by Larned and others (2010) in their “Punctuated Longitudinal Reactor” concept for temporary rivers. This concept states that *there are longitudinal gradients in POM processing rates due to repeated cycles of transport and retention*. In our model, we explored this idea by incorporating POM storage in lateral dry streambed fractions (most extensively found during no flowing periods in intermittent streams), and POM exchange between dry and wet streambed fractions along the network, which is a spatially explicit manner of considering those repeated cycles of transport and storage. Our simulations confirm this prediction. Indeed, mean POM reactivity, and thus the associated processing rates, decreases moving downstream (Figure 4). Longitudinal gradients of POM processing rates are also influenced by differential storage between wet and dry streambeds. We find that the lateral flux between wet and dry portions varies along the river network as a function of riparian phenology and flow regime (Figure 3). In headwater catchments, simulations show that the amount of POM stored on the dry streambed can be large but is mainly limited to the leaf-fall period, and the opportunity for POM degradation is small as POM is rapidly transported downstream. Thus, the synchronicity between riparian phenology and flow events is essential for understanding stream POM fluxes in the upper parts of the river network. If flood events are erratic, as simulated for the high lateral hydrological contraction (Figure S5), synchronicity decreases and POM accumulates for longer periods in the dry streambed.

The second prediction of the “Punctuated Longitudinal Reactor” concept states that *POM degradation is higher under wet than under dry conditions*. Based on that and on our synthesis of empirical studies (see Table S1), we imposed in our model the condition for POM decomposition to be lower in dry than in wet streambeds. Thus, the dry portion of the streambed behaves as sort of a “passive storage”, where POM can be transiently stored while undergoing slow degradation that does not significantly alter its reactivity. Our simulations show that this “passive storage” is especially important in headwater streams during high lateral contraction, becoming a POM source for stream communities after flow resumption (Acuña and Tockner 2010; del Campo and others 2021b). As catchment size increases, the stock of POM on the dry streambed decreases compared to that on the wet fraction, and thus, the average reactivity of exported POM decreases because processing rates are higher under wet conditions (Figure 3). Moreover, the fact that most POM is located in the wet streambed fraction in lowland areas indicates that transport dynamics are mostly related to the temporal patterns of stream discharge, and disconnected from the temporal pattern of leaf litter fall inputs. Thus, as shown for other riverine carbon pools such as DOM (Raymond and others 2016), hydrological flow is the main driver of the POM flux in large catchments.

### Lateral Hydrological Contraction as a Major Control of POM Export and Reactivity

High lateral hydrological contraction creates longer periods with high POM storage and little transport, but our simulations show that overall export of POM is enhanced at the fluvial network scale (Figure 4). Under low lateral contraction (i.e. low flow variability) almost all leaf litter entering the stream is processed along the river network. The small amount of POM that does reach the outlet (around 2%) shows low average reactivity as a consequence of the extensive decomposition experienced during its transport downstream. In contrast, under high lateral contraction, around 50% of the leaf litter inputs reach the network outlet, and show small changes in its average reactivity compared to initial values. This pattern emerges due to two interacting features: first, and most obvious, longer storage in the dry fraction increases the subsequent transport of barely degraded material after flow resumption. Second, high contraction is associated with a flashy hydrologic regime with few large events capable to effectively shunt POM downstream. This also aligns with the third prediction of the “Punctuated Longitudinal Reactor” which states that *processing efficiency increases with the number of transport, retention and processing cycles.* (Larned and others 2010). The low lateral contraction scenario is associated with more frequent events of POM exchange between the wet and the dry streambed, which results in an overall higher processing efficiency. However, it should be noted that in our set-up, which modulates hydrological contraction through different flow regimes, the number of cycles, their magnitude and the partition of POM storage between wet and dry, are tightly interrelated. The correlation among these three factors might be weaker in real stream networks where other sources of hydrological contraction are possible such as water abstraction (Allen and others 2020). However, we also argue that such correlation cannot be overlooked, and that the effect of the number of cycles can hardly be tested in isolation in real systems, emphasizing the need for a mechanistic model as the one presented here.

### The Pulse-Shunt-Storage Concept

**Figure 6:**
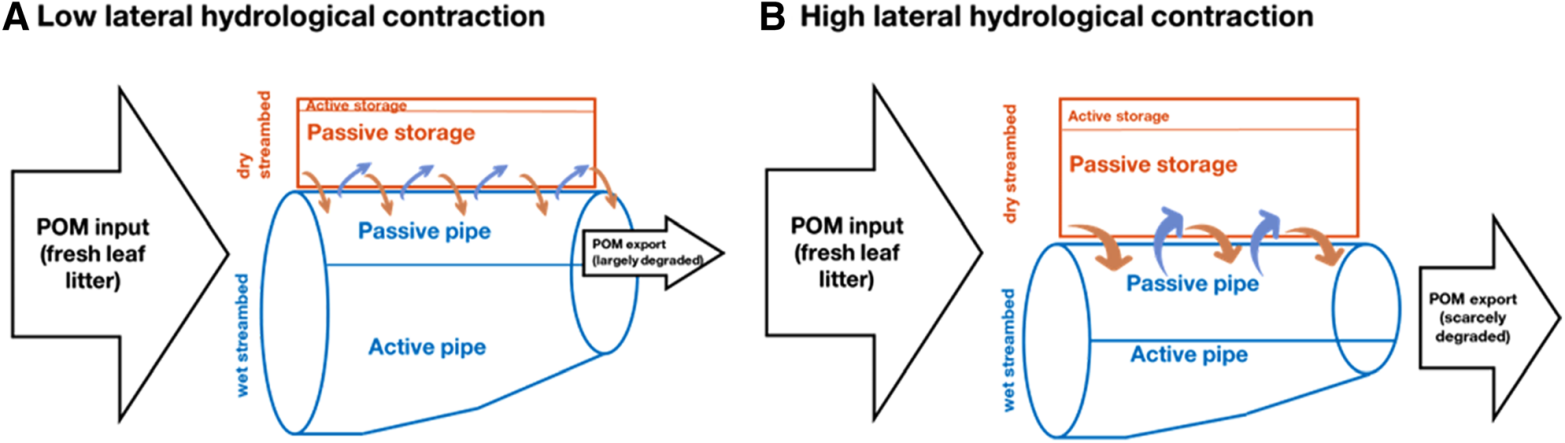
Conceptual representation of the “pulse-shunt-storage” modified from (Raymond and others 2016) for POM cycling in river networks under low (**A**) and high (**B**) hydrological contraction scenarios. This concept integrates the biogeochemical role of the dry fraction of the streambed on POM processing and transport. Terrestrial POM inputs as fresh leaf litter can reach the dry or wet fractions of the streambed. The POM entering the dry fraction is mainly stored until mobilized to the wet fraction (passive storage) but a small stock might be processed (active storage). (**A**) Under low hydrological contraction, there is a high number of exchange cycles between dry and wet fractions and POM is largely processed in the river network and only a small stock largely degraded is exported. (**B**) Under high lateral hydrological contraction, the role of the dry streambed expands, storage and stormy, fast transport of POM are favoured, POM is scarcely processed in the river network and a large stock of scarcely degraded POM is exported. Note that, under both hydrological conditions, export can only occur when POM is on the wet fraction.

Our model conceptualization, termed here “pulse-shunt-storage”, goes one step further from the passive versus active pipe dichotomy proposed in the “pulse-shunt concept” (Raymond and others 2016) by incorporating key ideas derived from the “punctuated longitudinal reactor” framework (Larned and others 2010). The pulse-shunt concept was developed to capture the effect of flow variability on DOM transport, but it does not consider POM, neither the impact of high hydrological contraction on organic matter cycling and transport. To understand POM cycling in a river network, the functions associated to POM storage and processing in dry streambeds need to be incorporated. Those functions might be particularly relevant in highly seasonal fluvial networks as stated by Larned and others (2010). This is why we have included a storage pool which can be either passive or active from a POM processing perspective (Figure 6). The passive storage refers to the function of dry streambeds of retaining POM while slowing down its degradation. Depending on POM composition, environmental conditions, and residence time, a non-negligible fraction of POM stored in the dry streambed can be decomposed, and we refer to this function as active storage. Therefore, our model contributes to expand the initial pulse-shunt concept by incorporating some features of dry streambeds, which impact on riverine POM processing such as: (1) characteristic sedimentation and re-suspension features, (2) spatio-temporal dynamics of lateral hydrological contraction and (3) the effect of dry streambed on POM degradation. Our results suggest that incorporating a dry streambed component in the pulse-shunt concept could be essential if we are to understand the storage and processing of POM across river networks, especially because many rivers experience some degree of lateral hydrological contraction (Figure 6).

Storms and the proportion of dry versus wet streambed area are linked in our conceptualization because scenarios of high flow variability where storms are frequent lead to increases in the streambed area and to larger fractions of dry streambed sediments in-between storms. Thus, high hydrological variability co-occurs with the increased relevance of both the shunt (i.e. passive pipe) and the passive storage effects on POM transport and processing. This is a differential feature compared to previous conceptual models of DOM processing at the network scale, which have related lower discharge with more extensive organic carbon degradation (Casas-Ruiz and others 2020; Creed and others 2015; Hotchkiss and others 2015; Raymond and others 2016; Bertuzzo and others 2017). Noteworthy, DOM does not occur in dry streambed fractions, and thus, its decay is directly linked with water residence time (Catalán and others 2016). POM, in contrast, becomes less dependent on water residence time due to its storage in dry fractions which becomes a central feature of the river network. Therefore, including the DOM fraction into the pulse-shunt-storage concept will enhance a) the active pipe effect (i.e. DOM will be mainly processed) under low hydrological contraction and b) the shunt effect, under high hydrological contraction scenarios (i.e. DOM will be mainly flushed downstream with little opportunity of being degraded). The differential influence of water residence time on either DOM or POM is particularly relevant when considering how flow variability is expected to vary in response to climate change (Schewe and others 2014; De Girolamo and others 2022). Overall, increased flow variability and hydrological contraction will translate in more exported of less degraded POM, which could impact the receiving ecosystems, with, for example, increased sedimentation of POM in coastal ecosystems, but also the functioning of the riverine food webs, with less POM available to be consumed in situ. Our simulations suggest that POM preconditioning, i.e. physicochemical processes occurring while POM is stored in dry areas which might alter its degradation after flow resumption (Abelho and Descals 2019; Mora-Gómez and others 2020; del Campo and others 2021b), has small relevance at the river network scale. According to our model, the accumulation of POM on the dry streambed (i.e. the passive storage) prevails over any decomposition enhancement of preconditioned leaf litter upon flow resumption (Figure 6). For instance, overall POM export was higher in the scenarios with relatively high degradation in dry streambed portions ($K_{\text{D}}/K_{\text{W}}$ = 0.3) (Figure 5). Even when considering higher degradation rates in dry streambeds, the effect of transport and lateral contraction prevails. Basically, flow interruption does not enhance the operation of the reactor as proposed in Larned and others (2010) but modifies the release of its inputs. However, note that longitudinal (for example, ponds formation) or vertical (for example, connection with the hyporheic zone) contraction not accounted in our model could influence POM reactivity by diversifying its sources (del Campo and others 2021a).

### Sensitivity Analysis, Model Limitations and Future Directions

The model presented here shows that hydrological contraction is key to driving POM export and reactivity, but other parameters are also influential, though to a lower extent. The reactivity distribution of fresh leaf litter is a major factor determining the share of POM either exported out or processed within the system, especially with increasing hydrological contraction (Figure 5). The river network can act almost as a passive pipe for POM when leaf litter reactivity is low and flow variability is high. At the other end, the higher the reactivity of POM, the highest the fraction that will be processed en route towards the sea. For instance, our model simulated a 20% decrease in export of POM when its average reactivity was high, independently of the degree of hydrological contraction, a result that matches well with the idea that leaf litter reactivity distribution is a major driver of organic matter degradation (Boyero and others 2016; Zhang and others 2019). Moreover, this result highlights the strong linkages between river networks and their riparian zones, catchment vegetation and land uses (Bormann and Likens 1967). In any case, regardless of having contrasting initial reactivity, hydrological contraction had a dominant influence on transport versus, processing POM patterns, enhancing higher export of less degraded POM export relative to its initial reactivity. Similarly, the low sensitivity of our model to the transport parameter (Figure 5), that controls the rate at which POM is transported downstream, indicates that the effect of lateral hydrological contraction on POM reactivity is independent of the transport capacity. One of the limitations of this model is the definition of bankfull width, which strongly influences the partitioning between wet and dry streambed. The bankfull width is based on a channel geometry in equilibrium with the assumed hydrological regime, which hampers us from disentangling the effects of flow variability versus lateral contraction (i.e. a more flashy hydrology also represents a larger streambed and dry fraction). However, we choose this model structure because by considering the same annual discharge across the three hydrological contraction scenarios, we were able to better understand the impact of lateral hydrological contraction on POM transport and export.

Our model was based on several assumptions that could be reconsidered in future exercises. First, different spatial drying patterns across fluvial networks could alter POM fluxes and their reactivity (del Campo and others 2021a). For instance, if hydrological contraction occurs mostly in headwaters, there will be increased export of large stocks of highly-reactive POM during posterior floods. Yet, if hydrological contraction is mostly constrained to lowland parts of the catchment, low export of POM stocks with very low reactivity might occur. Second, we used in-stream degradation rates of different tree leaf litter species reported in the literature to define the initial distribution of POM reactivities (summarized in Table S1) (Table 1). However, vegetation varies with changing flow variability and thus, obtained simulations might differ if different vegetation types are incorporated in the model. For example, higher flow variability might be linked to increased aridity, and therefore, to more xeric vegetation with less reactive leaf litter. Ephemeral desert streams would be an extreme situation, where minimum POM inputs of low reactivity would be expected. In contrast, deciduous trees (used in most experiments; see Table S1) are less likely to predominate in streams experiencing high flow variability conditions. So, we recommend future exercises to fine-tune vegetation/precipitation combinations. Note, also, that our results mainly apply to stream networks from sub-humid to temperate regions because we assume presence of significant riparian vegetation, and leaf litter fall peaking in autumn. As POM processing is linked to synchronicity between leaf litter fall and flow, we anticipate contrasting results in POM export dynamics in other biomes such as in tropical fluvial networks without seasonal leaf litter fall peaks. We assume leaf litter inputs occur homogeneously along the whole network. While this assumption might not be realistic in networks >3rd order, our model suggests that POM stocks in bigger catchments are mostly influenced by hydrology rather than by leaf litter inputs, and thus this assumption was fairly reasonable. Future exercises could consider other sources of POM than leaf litter, which might be relevant in specific climates or with higher levels of anthropogenic impact (for example, soil POM in agricultural catchments) (Tank and others 2018). The presence of dams or other human infrastructures could also impact flow regime and hydrological contraction patterns, thus influencing POM storage in dry riverbeds and its export downstream. Moreover, POM particle size might influence transport capacity through physical retention, and leaf litter is only a window in the full riverine organic matter continuum. Future model formulations could benefit from including either smaller or larger POM substrates (for example, fine detritus or wood), which account for a substantial fraction of terrestrial inputs (Datry and others 2018). A further step would be to simultaneously model POM and DOM (for example, Grandi and Bertuzzo 2022) in order to provide a more complete picture of C cycling and export in fluvial networks.

Flow variability significantly impacts the biogeochemical function of streams and rivers at the network scale through affecting the degree of lateral hydrological contraction. The mechanistic and conceptual model presented here (Figure 6) improves previous conceptualizations of riverine carbon cycling by explicitly incorporating the role of storage in dry streambed fractions. It opens exciting avenues to better test the behaviour of the POM pool along networks, and to understand the effect of changing runoff patterns and extreme events on the global role of rivers on the export and processing of terrestrial carbon from land to sea.

## Supplementary Information

Below is the link to the electronic supplementary material.

Supplementary file1 (PDF 3163 kb)

## Data Availability

Model code and simulations are available at https://doi.org/10.5281/zenodo.7142353
