# Supplementary material for: Pulse, Shunt and Storage: Hydrological Contraction Shapes Processing and Export of Particulate Organic Matter in River Networks
Source: Ecosystems. 2022 Dec 12;26(4):873–92. doi: 10.1007/s10021-022-00802-4 (PMC11420374; doi:10.1007/s10021-022-00802-4)
Supplement: Supplementary file 1 — Supplementary file1 (PDF 3163 kb) [file 10021_2022_802_MOESM1_ESM.pdf]

Hydrological contraction influences the dynamics of particulate organic matter at the river network scale
Supporting Information

Núria Catalán[a,b], Rubén del Campo[c,d], Matthew Talluto[c,d], Clara Mendoza-Lera[e], Giulia Grandi[f], Susana Bernal[g], Daniel von Schiller[h], Gabriel Singer[c,d], Enrico Bertuzzo[f]

[a]*Catalan Institute for Water Research (ICRA), Emili Grahit 101, 17003 Girona, Spain*
[b]*Laboratoire des Sciences du Climat et de l'Environnement, LSCE, CEA, CNRS, UVSQ, 91191, Gif-Sur-Yvette, France*
[c]*Department of Ecology, University of Innsbruck, Technikerstrasse 25, A-6020 Innsbruck, Austria*
[d]*Leibniz-Institute of Freshwater Ecology and Inland Fisheries (IGB), Müggelseedamm 310, 12587 Berlin, Germany*
[e]*Institute of Environmental Sciences, University of Koblenz-Landau, 76829 Landau, Germany*
[f]*Department of Environmental Sciences, Informatics and Statistics, University of Venice Ca' Foscari, 30170 Venice, Italy*
[g]*Integrative Freshwater Ecology Group, Center for Advanced Studies of Blanes (CEAB-CSIC), C/Accés Cala St. Francesc 14, 17300 Blanes, Spain*
[h]*Department of Evolutionary Biology, Ecology and Environmental Sciences, Faculty of Biology, University of Barcelona, Av Diagonal, 643, E-08028 Barcelona, Spain*

Table S1: Summary of the estimated parameters for the leaf litter degradation experiments

Reference	Species	Site	Sequential	ν [-]	$\langle K_{LF,W} \rangle$ [d^{-1}]	F_{WD} [-]	RMSE
Maamri et al. (2001)	willow	upstream	yes: D→W	11.00	0.0068	0.25	1.65
Maamri et al. (2001)	willow	downstream	yes: D→W	11.00	0.0054	0.11	1.65
Maamri et al. (1997)	willow	upstream	yes: D→W	2.74	0.0095	0.20	2.28
Maamri et al. (1997)	willow	downstream	yes: D→W	10.02	0.0072	0.114	3.11
Maamri et al. (1997)	nerium	upstream	yes: D→W	1.36	0.0080	0.20	2.99
Maamri et al. (1997)	nerium	downstream	yes: D→W	1.1	0.0099	0.09	2.01
Mariluan et al. (2015)	nothogafugs	intermittent stream	yes: D→W	0.34	0.0054	1.00	1.46
Mariluan et al. (2015)	nothogafugs	intermittent stream	yes: D→W	0.28	0.0068	1.00	2.27
del Campo et al. (2021)	reed	continental floodplain	yes: D→W	0.20	0.0324	0.11	1.20
del Campo et al. (2021)	reed	mediterranean floodplain	yes: D→W	0.20	0.0540	0.07	3.91
del Campo et al. (2021)	reed	arid floodplain	yes: D→W	11.00	0.0036	0.50	1.89
Battle and Golladay (2001)	cypress	FE wetland	yes: W→D	11.00	0.0028	1.00	4.00
Battle and Golladay (2001)	gum	FE wetland	yes: W→D	0.2	0.0518	0.97	5.78
Abril et al. (2016)	poplar	dry sediment + running waters	no	0.20	0.2300	0.05	2.04
Corti et al. (2011)	cottonwood	dry pools + wetpools	no	0.82	0.0656	0.10	1.85
Boulton (1991)	eucalyptus	upstream gravel + upstream river	no	0.53	0.0262	0.33	5.42
Boulton (1991)	eucalyptus	downstream gravel + downstream river	no	0.98	0.0196	0.40	6.64
Langhans et al. (2008)	poplar	gravel bar + river channel	no	1.40	0.0417	0.15	7.08
Langhans et al. (2008)	poplar	gravel bar + river channel	no	0.20	0.2589	0.063	4.48

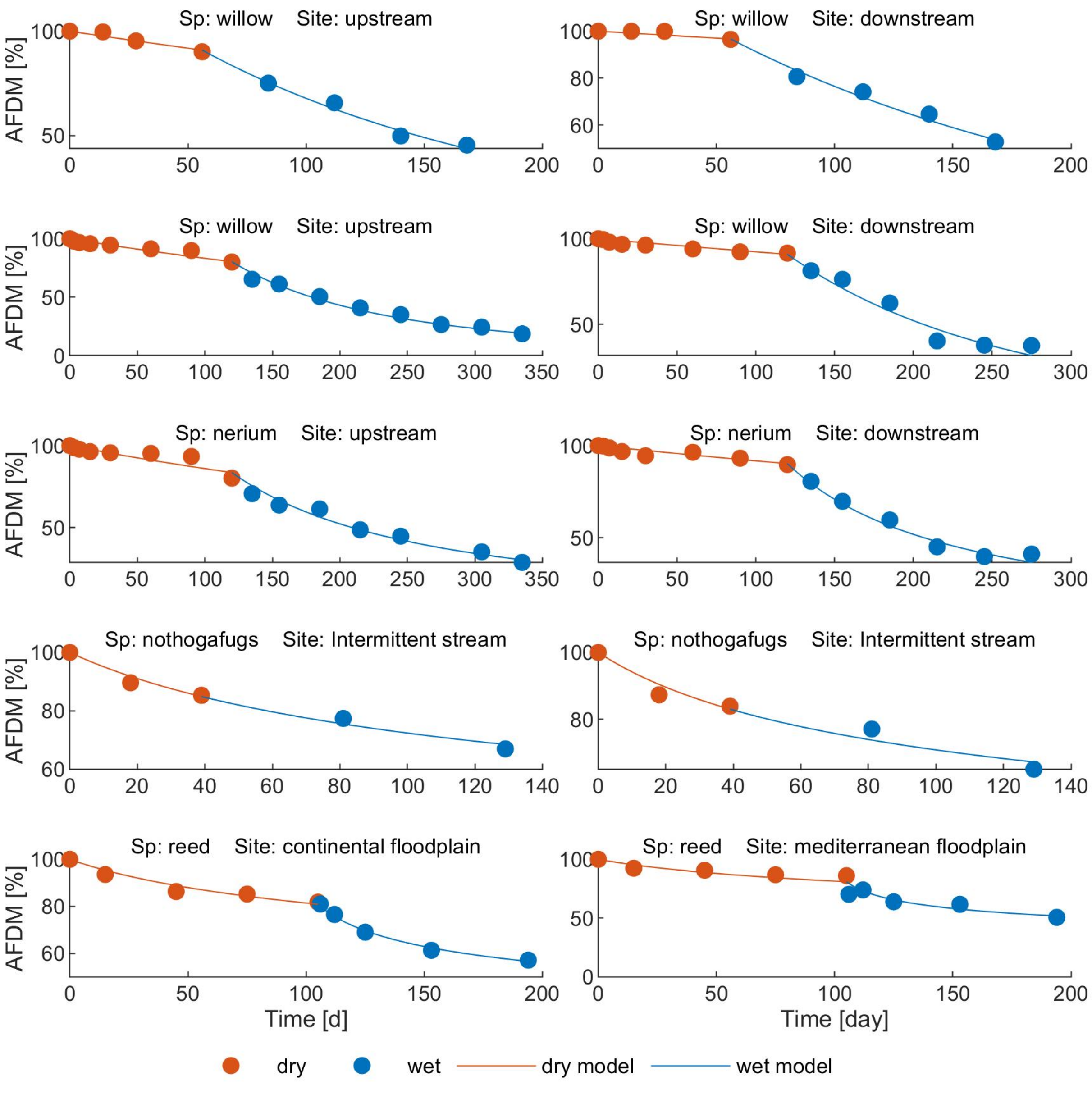

Figure S1: Fitting of RC model to experimental data. Sequential experiments.

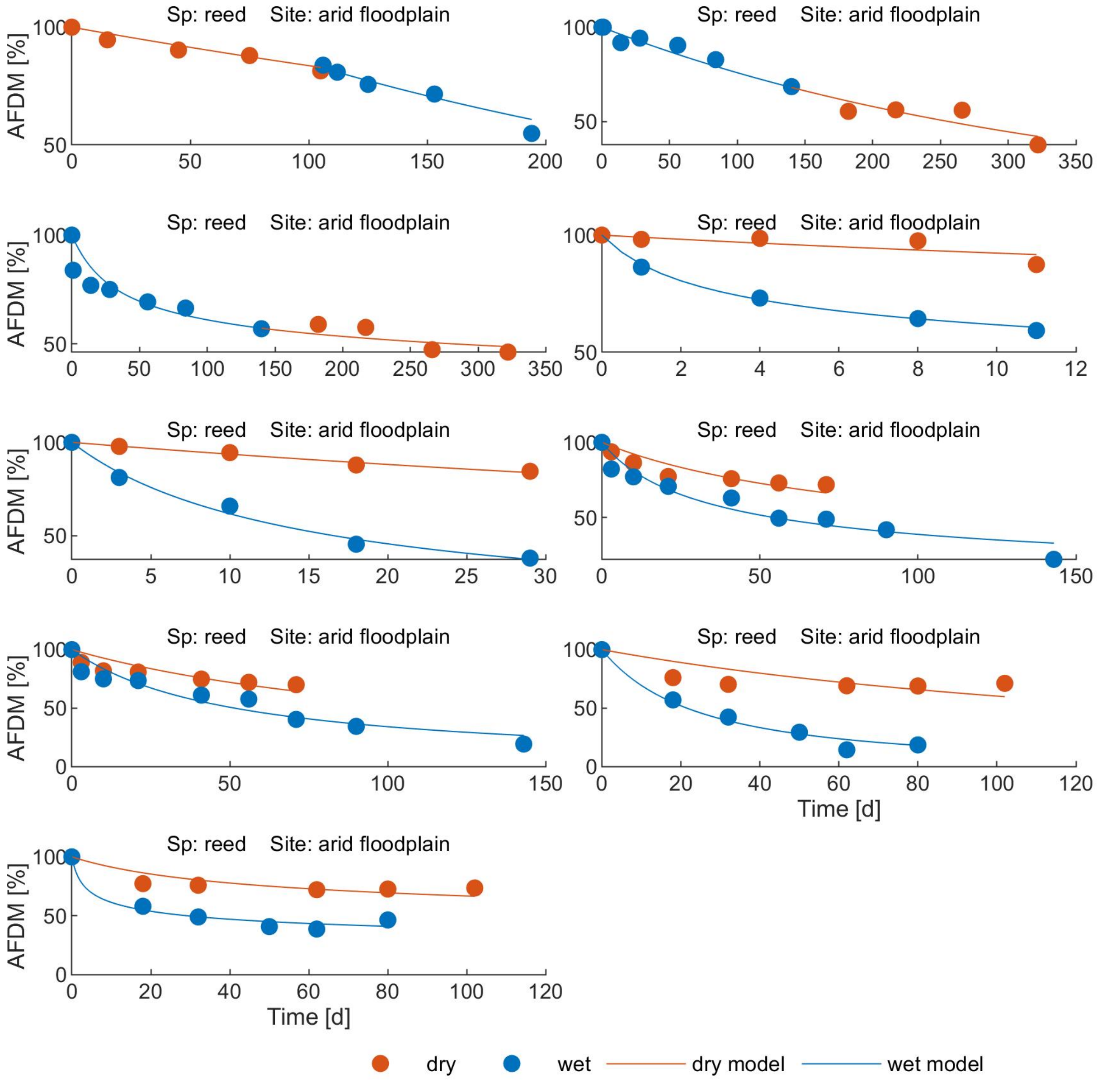

Figure S2: Fitting of RC model to experimental data. Sequential experiments (first three panels from top) and non-sequential ones.

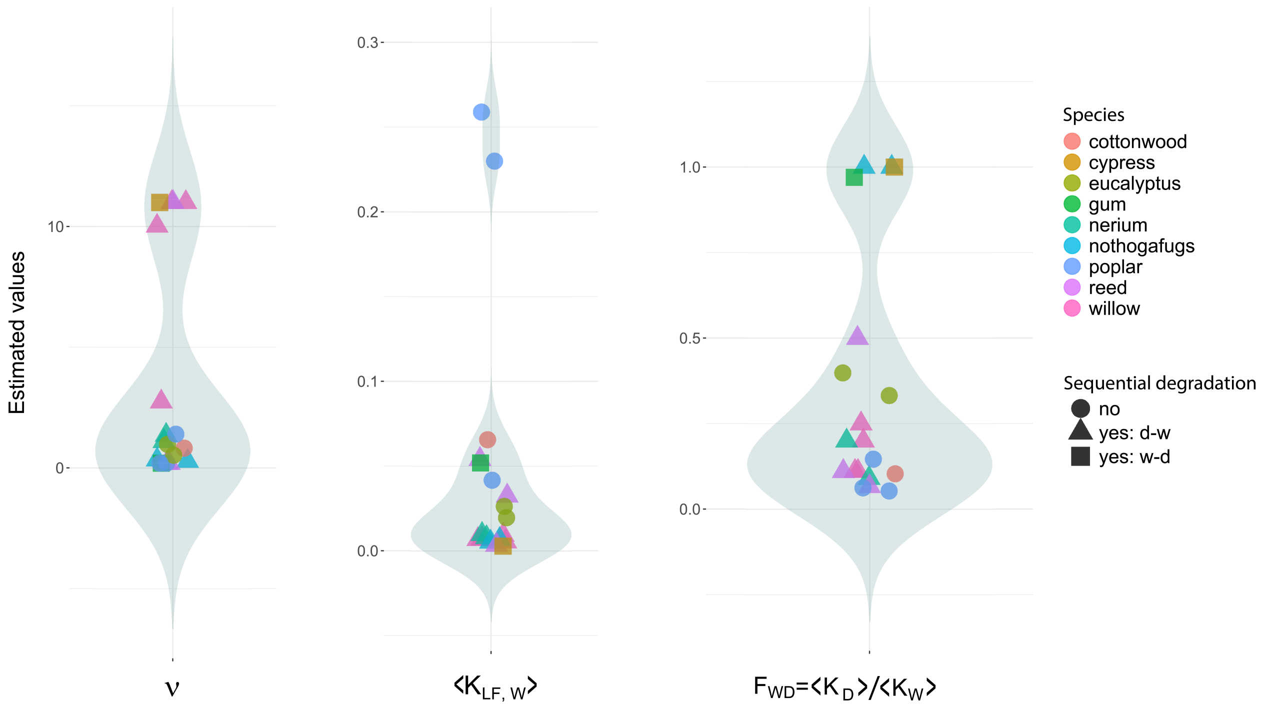

Figure S3: Summary of the estimated parameters

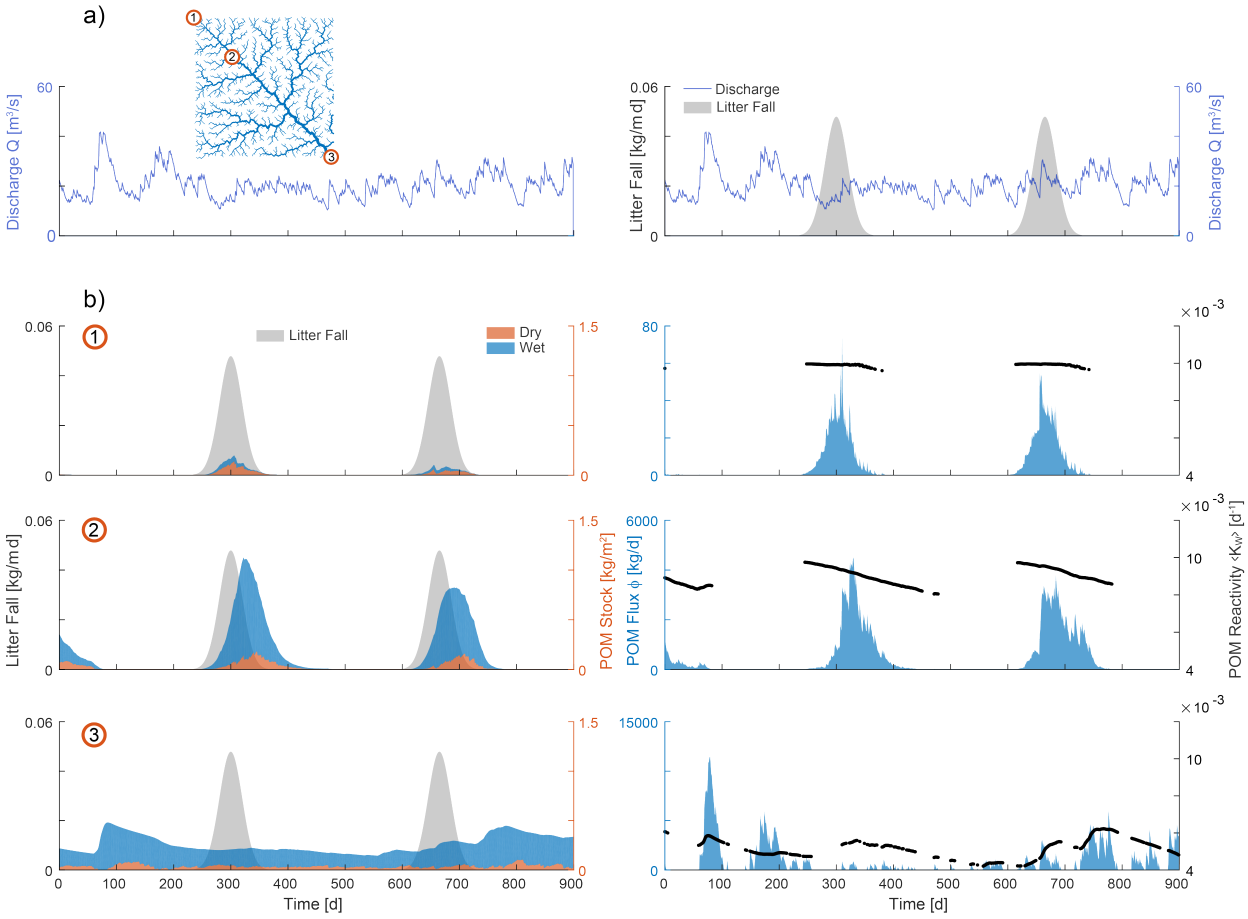

Figure S4: Same as Figure 3 but for the low lateral hydrological contraction scenario.

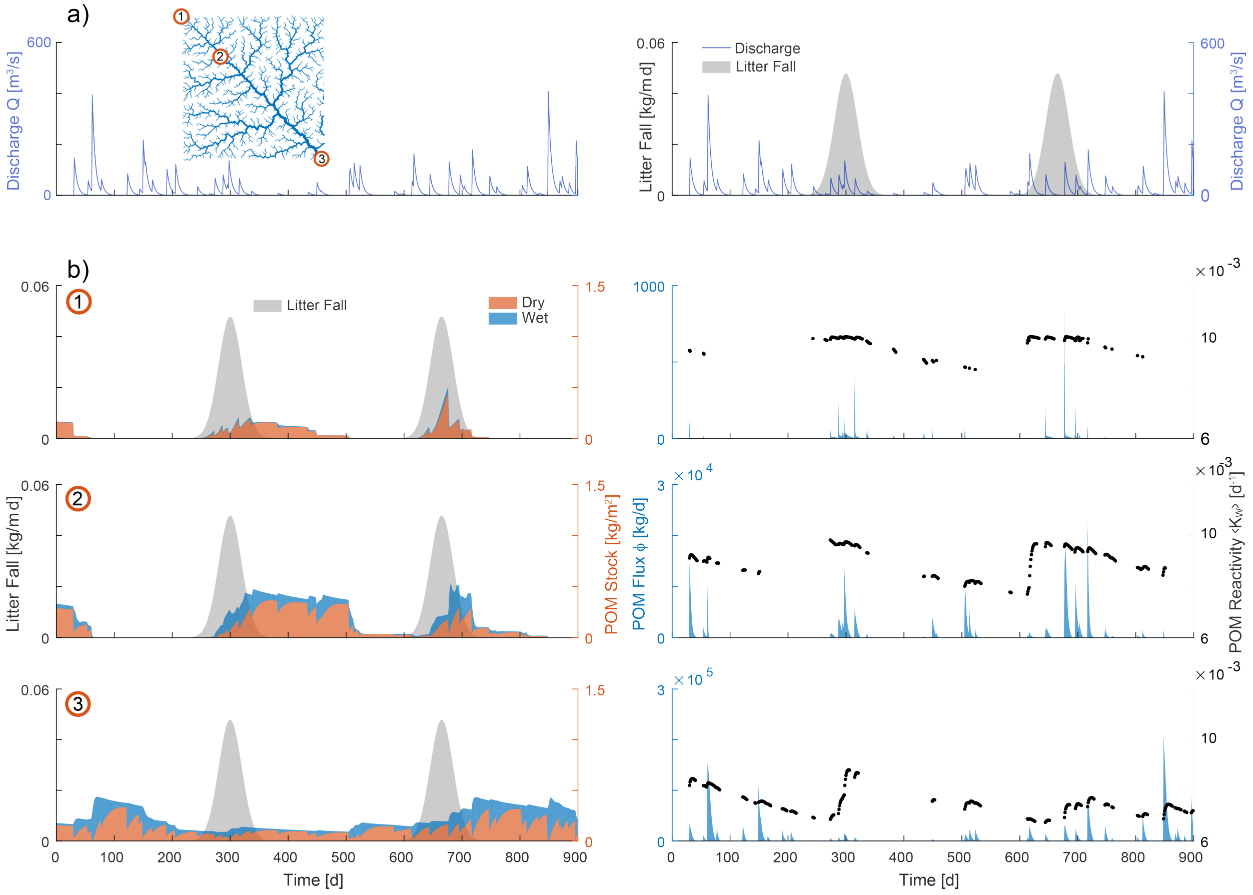

Figure S5: Same as Figure 3 but for the high lateral hydrological contraction scenario.

References

Abril, M., Muñoz, I., Menéndez, M., 2016. Heterogeneity in leaf litter decomposition in a temporary Mediterranean stream during flow fragmentation. Science of the Total Environment 553, 330–339. doi:https://doi.org/10.1016/j.scitotenv.2016.02.082.

Battle, J.M., Golladay, S.W., 2001. Hydroperiod influence on breakdown of leaf litter in cypress-gum wetlands. The American Midland Naturalist 146, 128–145. doi:https://doi.org/10.1674/0003-0031(2001)146[0128:HIOBOL]2.0.CO;2.

Boulton, A.J., 1991. Eucalypt leaf decomposition in an intermittent stream in south-eastern Australia. Hydrobiologia 211, 123–136. doi:https://doi.org/10.1007/BF00037368.

del Campo, R., Martí, E., Bastias, E., Barberá, G.G., Sanchez-Montoya, M.d.M., Gomez, R., 2021. Floodplain preconditioning of leaf litter modulates the subsidy of terrestrial c and nutrients in fluvial ecosystems. Ecosystems 24, 137–152. doi:https://doi.org/10.1007/s10021-020-00508-5.

Corti, R., Datry, T., Drummond, L., Larned, S., 2011. Natural variation in immersion and emersion affects breakdown and invertebrate colonization of leaf litter in a temporary river. Aquatic Sciences 73, 537–550. doi:https://doi.org/10.1007/s00027-011-0216-5.

Langhans, S.D., Tiegs, S.D., Gessner, M.O., Tockner, K., 2008. Leaf-decomposition heterogeneity across a riverine floodplain mosaic. Aquatic Sciences 70, 337–346. doi:https://doi.org/10.1007/s00027-008-8062-9.

Maamri, A., Bärlocher, F., Pattee, E., Chergui, H., 2001. Fungal and bacterial colonisation of salix pedicellataleaves decaying in permanent and intermittent streams in eastern morocco. International Review of Hydrobiology: A Journal Covering all Aspects of Limnology and Marine Biology 86, 337–348. doi:https://doi.org/10.1002/1522-2632(200106)86:3<337::AID-IROH337>3.0.CO;2-N.

Maamri, A., Chergui, H., Pattee, E., 1997. Leaf litter processing in a temporary northeastern moroccan river. Archiv für Hydrobiologie 140, 513–531. doi:https://doi.org/10.1127/archiv-hydrobiol/140/1997/513.

Mariluan, G.D., Díaz Villanueva, V., Albariño, R.J., 2015. Leaf litter breakdown and benthic invertebrate colonization affected by seasonal drought in headwater lotic systems of andean patagonia. Hydrobiologia 760, 171–187. doi:https://doi.org/10.1007/s10750-015-2324-z.
